# Nanometric Virus-Like Particles: Key Tools for Vaccine and Adjuvant Technology

**DOI:** 10.3390/vaccines8030430

**Published:** 2020-07-31

**Authors:** Giuseppe Bardi

**Affiliations:** Nanobiointeractions & Nanodiagnostics, Istituto Italiano di Tecnologia, Via Morego 30, 16163 Genova, Italy; giuseppe.bardi@iit.it; Tel.: +39-010-28-96-519

**Keywords:** vaccine, immune system, virus-like particles, nanoparticles

## Abstract

The ideal vaccine should trigger a specific response against pathogens and induce the immune system memory to be prepared for eventual following infections. Although different approaches to develop new vaccines are currently taken, several of the features of natural pathogens that allow a tailored immune reaction are difficult to mimic. The viral capsids are the physical interface between a virus and the host defense machinery which recognizes specific patterns of the viral supramolecular complexes. Therefore, empty viral particles deprived of their genomes represent optimal targets to induce immune reactions with several advantages for vaccination and adjuvant realization.

Viral genomes are shielded by proteins that are able to self-assemble in supramolecular complexes called capsids, which define the stability and infectivity of the virus. The review article by Mohsen and colleagues [1] describes the class of nanoparticles (NPs) derived from the viral capsids and their interactions with the immune system. The nanometric size and protein materials of the capsids suggest different potential biomedical applications. Actually, genome-deprived virus-like particles (VLPs) formed during infection were discovered decades ago. Their employment for human immunization has already allowed for the production of some approved vaccines and others in clinical trials. As expected, our immune system evolved by encountering diverse microorganisms and implemented their interception by recognizing particular structures. The authors exhaustively explain how the VLPs pathogen-associated structural pattern (PASP) can be identified by the innate immune system, eliciting a prompt response, otherwise provided by higher doses of artificial vaccine adjuvants. Furthermore, VLPs can be modified by molecular biology techniques or chemical crosslinking coupling heterologous antigens on the surface to increase their immunogenicity.

Although the absence of their own nucleic acid represents a modification of the original viral particle, VLP stability in biological fluids permits their migration to the draining lymph nodes (LNs). T and B lymphocytes are anatomically segregated in the different areas of the LN. This is an important point to properly design a VLP-based vaccine since the immune response will be influenced by the VLP localization within the LN. Antigens that enter the LN subcapsular sinus are sorted according to their size. Nanometric size particles like viruses (20–200 nm) that freely move in the lymph can be taken up by sinus macrophages and presented to cortical B lymphocytes underneath the sinus. However, specialized dendritic cells (DCs) can mature by capturing VLPs in the peripheral tissues. Differentiated DCs express the same chemokine receptor, CCR7, that allows the naïve T lymphocytes to localize into the LN T cell zone. This process is driven by the gradient of the chemokines CCL19 and CCL21, both binding CCR7. The VLP processed peptides presented by DCs to the T cell receptor stimulate a cellular mediated immune response. So, both the B cell-dependent humoral response and the T cell-dependent cellular mediated immune response can be triggered by VLPs, increasing their potential for human immunization.

The amplification of the immune response required to raise the efficiency of a vaccine is mediated by the communication between the innate and adaptive immune system, precisely by the inflammatory cytokine exchange between monocyte/macrophage phagocytes and CD4^+^ T helper lymphocytes. Together with pattern recognition receptors (PPRs) distinguishing several conserved structures of microorganisms, antigen phagocytosis is accelerated by molecules that opsonize pathogens and bind them to specific membrane receptors. IgM antibodies or pentraxins are examples of such molecules able to bind VLPs and activate an enzymatic cascade of the complement proteins, leading to particle internalization. Together with the VLP peptides’ processing and presentation to CD4^+^ T lymphocytes, monocyte/macrophage phagocytes initiate the transcription and release of inflammatory cytokines acting on other immune cells in a feedforward amplification mechanism. In addition, Mohsen at al. report the long-lasting humoral response evoked by VLPs, underlining how their organized and highly repetitive structure can facilitate the B cell receptor (BCR) crosslinking, surpassing the B lymphocyte activation threshold and the initial T-helper lymphocyte-mediated cytokine support.

The humoral response to foreign antigens is also characterized by antibody switching. This process is dependent on the endosomal toll-like receptor seven (TLR7) and TLR9 which usually bind single strand RNAs (ssRNA) or DNA unmethylated CpG motifs, respectively. VLPs can be loaded with particular nucleic acids in order to modulate a precise immune response. Though this feature emphasizes the potential of VLPs in advanced vaccine preparation, it expands the biomedical applications of these NPs as drug or gene delivery systems. The immune response towards VLP-delivery carriers should be considered and their immunogenicity must be reduced instead of increased, for example by surface modifications with immune-stealth polymers (e.g., PEG (Polyethylene glycol)) or soluble proteins (e.g., albumin).

Other interesting points are discussed in the review, such as the efficient presentation of VLP derived peptides by both MHC I (CD8^+^ T cell restricted) and MHC II (CD4+ T cell restricted) molecules, or the PPR activated signal transduction pathways leading to inflammatory cytokine release. However, the authors do not limit the discussion to the positive aspects of using VLPs in vaccine development, but conclude the review considering some important limitations. First of all, the high immunogenicity obtained by functionalizing VLP surfaces with heterologous antigens using chemical crosslinking or genetic fusion. The choice of the method affects the VLP stability and the quality of the immune response. A second examined limitation depends on the host expression system. Randomly packed components of the host during VLP assembly could have unknown effects and compromise the scaled up production of a vaccine. In conclusion, low stability of recent VLP-based vaccines and the absence of surface modified VLPs with foreign epitopes are mentioned.

The review offers a valid overview of the VLP interactions with the immune system and their potential for future biomedical applications.
